# Three-Year Results Following Microwave Therapy in Patients with Severe Primary Axillary Hyperhidrosis

**DOI:** 10.1007/s00266-025-05469-5

**Published:** 2025-12-01

**Authors:** Emanuela Micu, Maria Fragkou Dragka, Alexander Shayesteh

**Affiliations:** 1 Department of Dermatology and Venereology in Östergötland, and Department of Biomedical and Clinical Sciences, Linköping University, Linköping, Sweden; 2 Department of Public Health and Clinical Medicine, Dermatology and Venereology, Umeå University, Umeå, Sweden

**Keywords:** Hyperhidrosis, Microwave, Miradry^®^, Quality of life

## Abstract

Microwave therapy (Miradry^®^) is an approved treatment for axillary hyperhidrosis (AH). There are several studies in the literature that show favourable safety and efficacy profile, although a few follow up patients under longer period and on larger cohort patients. In the present study, we report three-year results after microwave therapy for AH. At dermatology clinic in Östergötland 103 patients with severe AH received one or two Miradry^®^ treatments, between 2020 and 2022. Patients were examined at several intervals during study period. Between March 2024 and June 2025, 87 patients were contacted by post and asked to complete HDSS (Hyperhidrosis Disease Severity Scale) and Hyperhidrosis Quality of Life (HidroQoL^©^); 45 patients have responded to our survey (response rate 51.7%). Statistically significant improvement was observed in both HDSS (from medians 3 at the study inclusion to medians 2 at 3 year) and HidroQoL^©^ (medians 26 at baseline and medians 6 at 3 year). As a conclusion, our data demonstrate that microwave therapy is a promising long-term efficient treatment for AH and significantly improves quality of life in patients suffering from severe AH.

*Level of Evidence II* This journal requires that authors assign a level of evidence to each article. For a full description of these Evidence-Based Medicine ratings, please refer to the Table of Contents or the online Instructions to Authors www.springer.com/00266.

## Introduction

Non-invasive microwave thermolysis of axillary sweat glands using Miradry^©^ device (Miramar Labs, Sunnyvale, CA) is an approved treatment for axillary hyperhidrosis (AH), used worldwide for more than a decade. It is performed under local tumescent anaesthesia using a machine with a handpiece that emits microwaves at 5.8 GHz fixed frequency, a cooling system that prevents damage to the superficial skin layers, and a vacuum system that lifts the underlying skin to help isolate the target tissue from underlying structures. In theory, this method could provide long-lasting effect for AH, as the sweat glands are not expected to regenerate.

AH is a dermatological condition associated with social and emotional stress, affecting health-related quality of life (QoL) [1, 2]. Measuring AHs impact on QoL can be performed by using patient-reported outcome measures (PROMs). Using PROMs enables the physician to tailor individual treatment and grants access to more expensive treatments that can provide long-lasting relief. Most of these therapies (among which is microwave thermolysis) are available only in hospitals or private clinics thus highlighting the importance of proper assessment.

Several studies have shown a high success rate of Miradry^®^, with 89–90% of treated patients, demonstrating improvements based on Hyperhidrosis Disease Severity Scale (HDSS) score [3]. Fewer trials have long-term monitored the microwave therapy for hyperhidrosis with follow-up periods of one to five years [4–6]. These studies, however, showed low response feedback from participants, and most of them have included small groups of patients.

We previously reported promising results of Miradry^®^ on AH at 1-year follow-up [7]. The aim of this study is to evaluate the long-term prognosis of microwave therapy at 3-year follow-up. Assessment was based on the classical HDSS but also on a newer, validated hyperhidrosis-specific questionnaire, Hyperhidrosis Quality of Life (HidroQoL^©^). The HidroQoL^©^ has showed sensitivity to symptom improvements at several measurement timepoints, proving its longitudinal validity [8].

## Materials and Methods

### Patients

**Fig. 1:**
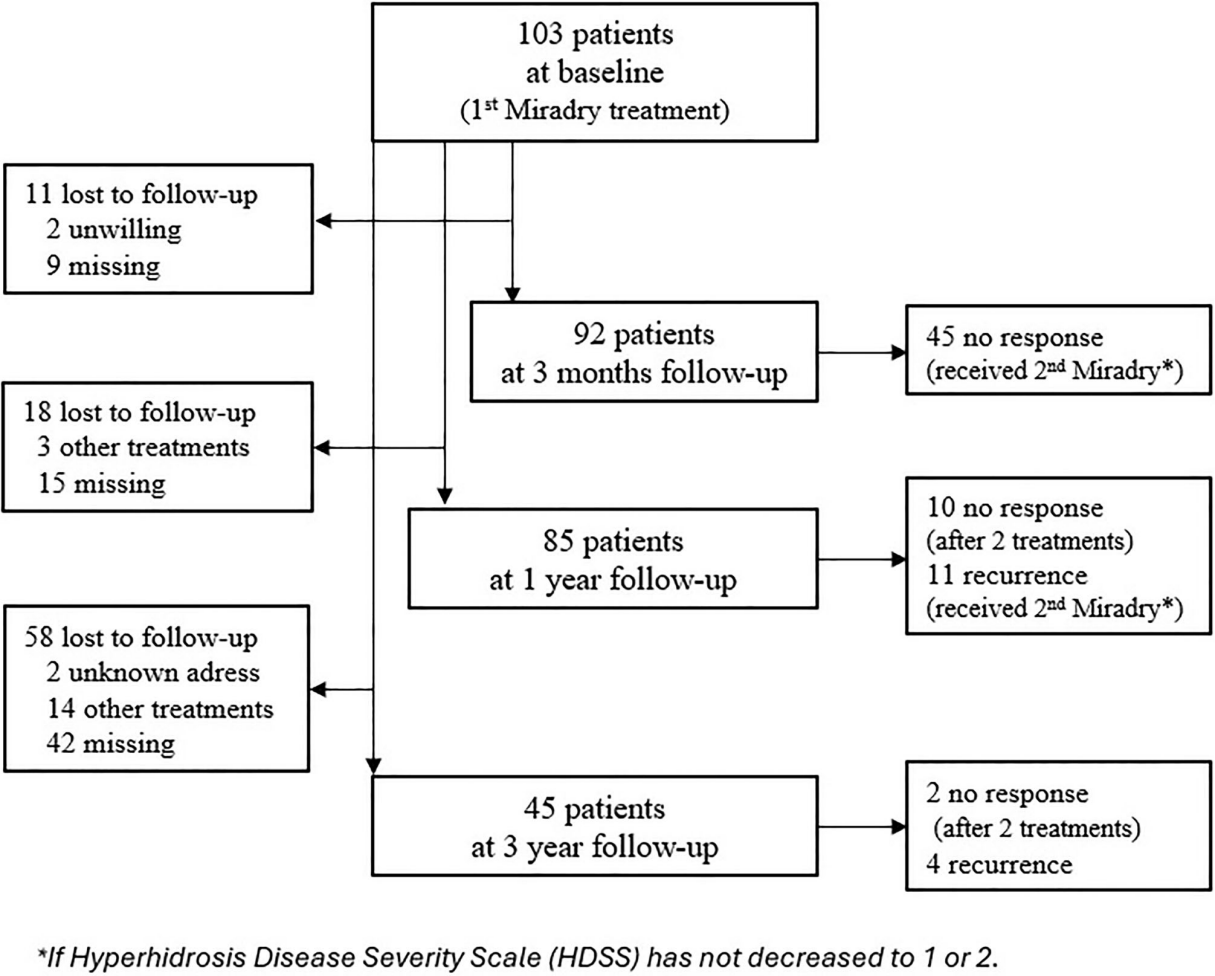
Patients flow chart

At the dermatology department at Vrinnevi Hospital, Norrköping, Sweden, a total of 103 patients (74% female and 26% male patients) were treated using Miradry^®^ between September 2020 and June 2022 as part of our previous study. Criteria for recruiting the patients and the procedure were described in detail elsewhere [7]. Only patients with severe AH defined as HDSS of 3 or 4 were included in the study, and no other hyperhidrosis treatments were allowed during study period except for over-the-counter antiperspirants. Patients were evaluated at 3 months and 1 year and received a second treatment if their HDSS score had not decreased to 1 or 2. Of 103 patient, 87 were asked to take part in a questionnaire survey, between March 2024 and June 2025, at least 3 years after the first Miradry^®^ treatment. (Two patients were not reachable because of unknown address, and 14 patients were not eligible because they have received other hyperhidrosis treatments since the last Miradry^®^ session.) Treatment history and background data were collected from patients’ medical records according to study plan. See Fig. 1 for study flow chart.

The study was performed in compliance with the Declaration of Helsinki and International Council for Harmonisation guideline of Good Clinical Practice. Study was approved by the regional ethics committee (approval no. # 2019-06052 and no # 2023-06023-02). Informed written consent was obtained from study patients prior to inclusion and for the follow-ups. The study was registered at ClinicalTrials.gov (NCT04546438).

### Questionnaires

Participants symptoms were assessed using PROMs such HDSS and HidroQoL^©^. PROMs were collected at baseline, 1-year, and 3-year follow-ups.

HDSS consists of four statements about the impact of sweating on daily life, scored from 1 to 4 points. The score increases according to the severity of the disease. An HDSS score of 1 corresponds to no or mild disease, 2 points mean moderate disease, and 3 or 4 points constitute severe hyperhidrosis.

HidroQoL^©^ is a validated hyperhidrosis-specific questionnaire with two impact domains: these are Daily Life Activities (6 items) and Psychosocial Life (12 items) domains (with total max score 36) [9]. The higher the total score, the more severe the impact of hyperhidrosis is on the patient’s life. A recent study has proposed a score banding system such as 0–6 (no effect), 7–18 (small effect), 19–25 (moderate effect), 26–32 (very large effect), and 33–36 (extremely large effect) on a person’s QoL [10].

### Statistics

PROMs were categorized as ordinal data, and a nonparametric test for paired data was applied (Wilcoxon’s signed-rank test), which was presented with medians. *P*-values <0.05 were considered statistically significant, “*p*” values representing comparison of baseline and follow-ups. Statistical analysis was performed using IBM SPSS Statistics (version 30.0.0.0; IBM Corp, Armonk, NY, USA).

## Results

In the following, the results of 1-year and 3-year surveys are depicted to assess long-term effect of Miradry^®^. Baseline characteristics, side effects, and 3-month results were analysed in detail in our previous study [7]. We included 103 patients at baseline, 76 female (73.7%) and 27 men (26.2%); at one year 85 patients, 63 female (74.1%) and 22 male patients (25.8%); at 3 years 45 patients, 33 female (73.3%) and 12 male patients (26.6%). Figure 1 shows study flow and patients’ flow.

**Fig. 2:**
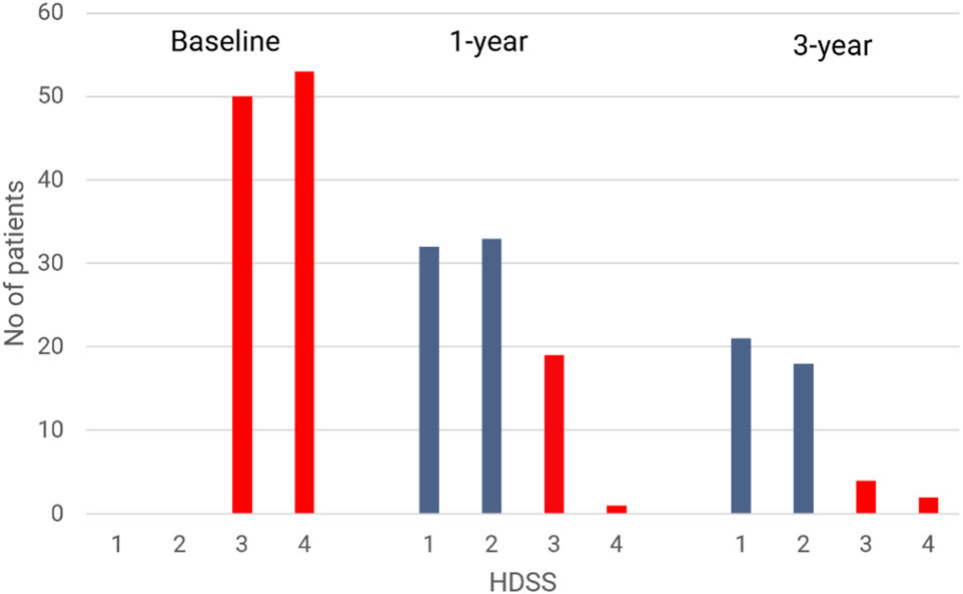
Hyperhidrosis Disease Severity Scale (HDSS) at baseline and follow-up visits. HDSS scale: 1, sweating never noticeable and never interferes with daily activities; 2, sweating tolerable but sometimes interferes with daily activities; 3, sweating barely tolerable and frequently interferes with daily activities; 4, sweating intolerable and always interferes with daily activities. Patients received Miradry^®^ at baseline and in patients that did not obtain HDSS 1 or 2 a second treatment was indicated in the first year

At baseline, the HDSS median score was 3 and decreased during the follow-ups (medians 2 at 1 year and at 3 years) (Fig. 2). At the three-year follow-up, 39 of 45 patients had HDSS score of 1 or 2 (86.6% response rate); of those, 21 patients had received two treatments and 18 patients one treatment during the first year. Six patients had HDSS score of 3 or 4 (13.3% response failure) at 3-year follow-up: patient 1 (female) lost the initial effect and did not answer after the second treatment either, and had a known depression diagnosis; patient 2 (male) did not have effect after the first not either after the second treatment, and had also depression diagnosis; patient 3 (male) had good effect after two treatments at one-year follow-up, but lost the effect at 3-year follow-up; three patients (all females) lost the effect at 3-year follow-up compared with one-year follow-up, and they had received only one treatment.

**Fig. 3:**
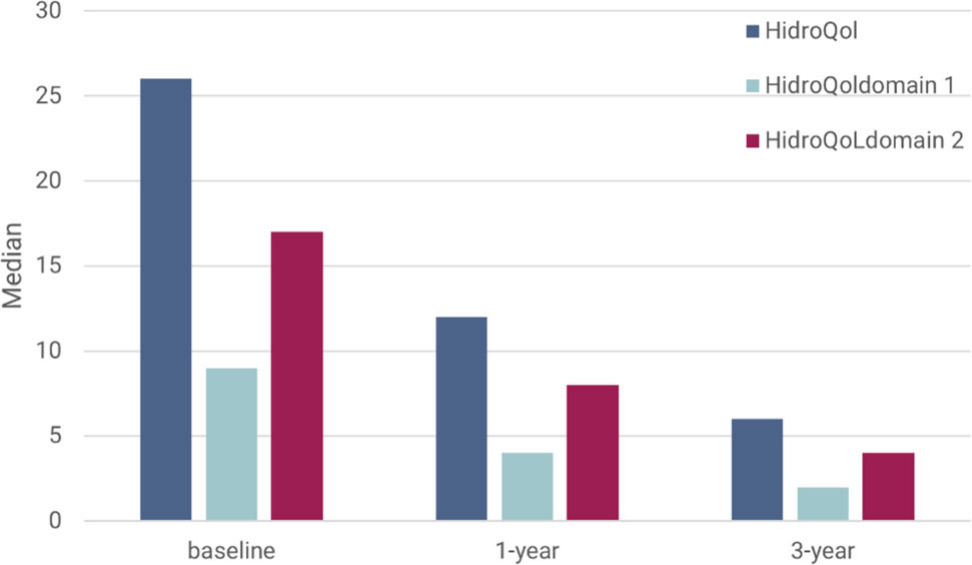
Hyperhidrosis Quality of Life Index (HidroQoL^©^) at baseline and follow-up visits. HidroQoL has two impact domains: domain 1, Daily Life Activities (6 items, ranges from 0 to 12) and domain 2, Psychosocial Life (12 items, ranges from 0 to 24). The total score is computed based on all the 18 items and ranges from 0 to 36. Patients received Miradry^®^ at baseline, and in patients that did not obtain HDSS 1 or 2, a second treatment was indicated in the first year

HidroQoL^©^ identified severe impact on QoL at baseline (HidroQoL^©^ median score 26 p) and significant improvement on the follow-ups: 1 year (median 12 p) and 3 years (median 6 p), *p*<0.001. Both domains of HidroQoL^©^ were improved (Fig. 3).

Table 1 shows outcome measures, at baseline, one, and three-year follow-ups. Table 2 shows comparison between one-year and three-year follow-ups, with improvement in the outcomes although not statistically significant.

**Table 1:** Differences in patient-reported sweat reduction compared with baseline

Questionnaire	N	Median	1^st^ Quartile	3^rd^ Quartile	Improved compared to baseline n (%)	No Change compared to baseline n (%)	Worsened compared to baseline n (%)	*P*-value
HDSSVisit1	103	3.00	3.00	4.00				
HDSSVisit2	85	2.00	1.00	2.00	75 (88.2)	10 (11.8)	0 (0.0)	<0.001
HDSSVisit 3	45	2.00	1.00	2.00	41 (91.1)	4 (8.8)	0 (0.0)	<0.001
HidroQolVisit1	103	26.00	21.00	31.00				
HidroQolVisit2	85	12.00	4.00	21.50	79 (92.9)	2 (2.4)	4 (4.7)	<0.001
HidroQolVisit3	45	6.00	2.00	16.00	42 (93.3)	1 (2.2)	2 (4.4)	<0.001

Visit 1 baseline when patients received first Miradry treatment, visit 2 is one year after the first Miradry treatment, visit 3 is three years after first Miradry treatment. *HDSS* Hyperhidrosis Disease Severity Scale, *HidroQoL* Hyperhidrosis Quality of Life Index

**Table 2:** Differences in patient-reported sweat reduction between first and third year of follow-up

Questionnaire	N*	Improved compared to visit 2 n (%)	No Change compared to visit 2 n (%)	Worsened compared to visit 2 n (%)	*P*-value
HDSSVisit3	43	9 (20.9)	25 (58.1)	9 (20.9)	*p*>0.05
HidroQolVisit3	43	18 (41.8)	10 (23.2)	15 (34.8)	*p*>0.05

^*^N number of patients with both visits for Wilcoxon test

Visit 2 is one year after the first Miradry treatment, visit 3 is three years after first Miradry treatment. *HDSS* Hyperhidrosis Disease Severity Scale, *HidroQoL* Hyperhidrosis Quality of Life Index

## Discussion

The results of our study demonstrated sustained effect over time for Miradry^®^ therapy, at three-year follow-up. The outcome improvement at three-year compared with one-year follow-up suggests that two therapy sessions may be more efficient than one, as there was recurrence of AH in some patients during the first year and they had received two Miradry treatments accordingly. However, in very few there was no response to two treatments, in which we can only speculate possible causes.

Nonresponse to destructive, minimal or non-invasive, procedures exist in the literature [11]. One explanation can be various localizations of different types of sweat glands. Generally, the gland responsible for axillary sweat is considered the eccrine glands, but in AH apocrine glands may play a role [12]. A third type of sweats gland had been described, the apocrine gland [13] with much higher capacity to produce sweat than eccrine glands and present abundantly in the axillae with possible implication in hyperhidrosis [14]. However, their existence is controversial and Bovell et al. [15] stipulate that higher sweat excretion is due to different levels of activation of sweats glands or susceptibility of activation. According to the study of Beer et al. [14], the sweat glands location is mostly in the subcutaneous tissue, near the border to the dermis, although classically it was considered to be situated in the deep dermis. The group of Lonsdale [16] has shown that both apocrine and eccrine glands are in close apposition and do not occupy different layers within the axillary skin; sweat glands started at a mean depth of 2.4 mm and stopped at the bottom of the excised skin specimen. (An average 3.5 mm of skin was occupied by glandular tissue.) [16].

The microwaves from Miradry^®^ penetrate to a specific depth between dermis and subcutaneous tissues regardless of skin thickness, with peak absorption at approximately 0.25 mm from dermal–hypodermal interface [17]. Consequently, in theory, all sweat glands within the targeted region should be ablated by Miradry^®^. A recent study confirmed a reduction in sweat glands following microwave thermolysis, although some atypical apocrine glands were still identified [18]. Other authors have speculated that the relapse of excessive sweating can be caused by incomplete heat damage of the dilated or deep secretory eccrine glands and large nerve fibres [19].

Microwaves are directed specifically in the axilla according to a template size selection with pre-established sizes, from 50 × 100 to 160 × 80 mm. Positioning of templates may leave certain areas with active eccrine sweat glands outside the treatment field. In this sense, a second treatment can target slightly different areas than the first by adjusting the positioning of the template and in some cases by choosing another template size, thus increasing the overall effect.

It is unclear whether previous Miradry^®^ treatment could have altered the sweat glands in the axillae, by promoting fibrosis that potentially leads to a diminished effect of a subsequent Miradry^®^ session [20], as in one of our patients who did not obtain the same effect of the second treatment compared with the first treatment. Further studies are needed to evaluate whether such tissue changes impact retreatment outcomes and to determine optimal strategies for template positioning and session planning to enhance efficacy.

### Strengths and Limitations

The main strengths of our study were the long term of follow-up period and a relatively large sample size to achieve statistically significance. Although almost half of the patients did not respond to this survey, hospital medical records indicate that they did not contact our clinic for worsening hyperhidrosis symptoms during the follow-up period. However, we could not establish if the patients who did not respond to our survey received other treatments from other clinics.

While our study demonstrates sustained improvement in HDSS and HidroQoL^©^ scores up to three years after Miradry^®^ treatment, it is important to acknowledge that these outcomes are not directly compared with other therapeutic options for AH. Alternative interventions, such as topical antiperspirants, botulinum toxin injections, and surgical procedures, also provide varying degrees of symptom relief with different durations of effect, invasiveness, and risk profiles. Direct comparisons of long-term efficacy, safety, and patient satisfaction between these treatments remain limited in the literature.

Some other limitations of our study were lack of objective methods to assess sweat production. Swedish guidelines do not stipulate to use objectively methods in hyperhidrosis evaluation. Moreover, objective measurements are usually performed at one time point and not considering real-life data.

In conclusion, three years after the treatment, our study shows that most surveyed patients did preserve a good response to the treatment. However, it may be necessary to recommend at least two treatments in some patients due to recurrences.

Our results could give guidance to healthcare professionals in choosing appropriate therapy for hyperhidrosis and recommend using novel, validated tools when evaluating hyperhidrosis impact on QoL.

As cost-effectiveness data are of more and more of interest in real world, clinical trials with larger patient groups along with long-term monitoring are mandatory to evaluate and compare Miradry^®^ with other available treatments.
